# On the Host Side of the Hepatitis E Virus Life Cycle

**DOI:** 10.3390/cells9051294

**Published:** 2020-05-22

**Authors:** Noémie Oechslin, Darius Moradpour, Jérôme Gouttenoire

**Affiliations:** Division of Gastroenterology and Hepatology, Lausanne University Hospital and University of Lausanne, CH-1011 Lausanne, Switzerland; Noemie.Oechslin@unil.ch (N.O.); Darius.Moradpour@chuv.ch (D.M.)

**Keywords:** HEV, host factor, particle production, viral replication, virus entry

## Abstract

Hepatitis E virus (HEV) infection is one of the most common causes of acute hepatitis in the world. HEV is an enterically transmitted positive-strand RNA virus found as a non-enveloped particle in bile as well as stool and as a quasi-enveloped particle in blood. Current understanding of the molecular mechanisms and host factors involved in productive HEV infection is incomplete, but recently developed model systems have facilitated rapid progress in this area. Here, we provide an overview of the HEV life cycle with a focus on the host factors required for viral entry, RNA replication, assembly and release. Further developments of HEV model systems and novel technologies should yield a broader picture in the future.

## 1. Introduction

Hepatitis E virus (HEV) has been identified as a cause of the waterborne hepatitis outbreaks in the early 1980s [1,2]. The viral genome was cloned and sequenced in 1990, allowing the development of serological tests to study its epidemiology [3,4]. The virus has been classified in the *Hepeviridae* family, and most human pathogenic strains belong to species Orthohepevirus A [5]. Members of this species can be classified into 8 genotypes (gt): gt 1 and 2 are restricted to humans and are transmitted via the fecal-oral route, mainly through contaminated drinking water. Gt 3 and 4 cause zoonotic infections and the transmission occurs mainly via the consumption of un(der)cooked pork, wild boar or deer meat. Gt 5 and 6 are found in wild boar, and gt 7 as well as 8 infect dromedary and Bactrian camels, respectively. Gt 7 has been identified in an immunosuppressed patient after consumption of camel milk and meat [6] (reviewed in [7]) but no transmission to humans has thus far been reported for gt 5, 6 and 8. More recently, rabbit HEV (closely related to gt 3 within species Orthohepevirus A) and rat HEV (belonging to species Orthohepevirus C) have also been found to infect humans [8,9,10,11].

**Figure 1:**
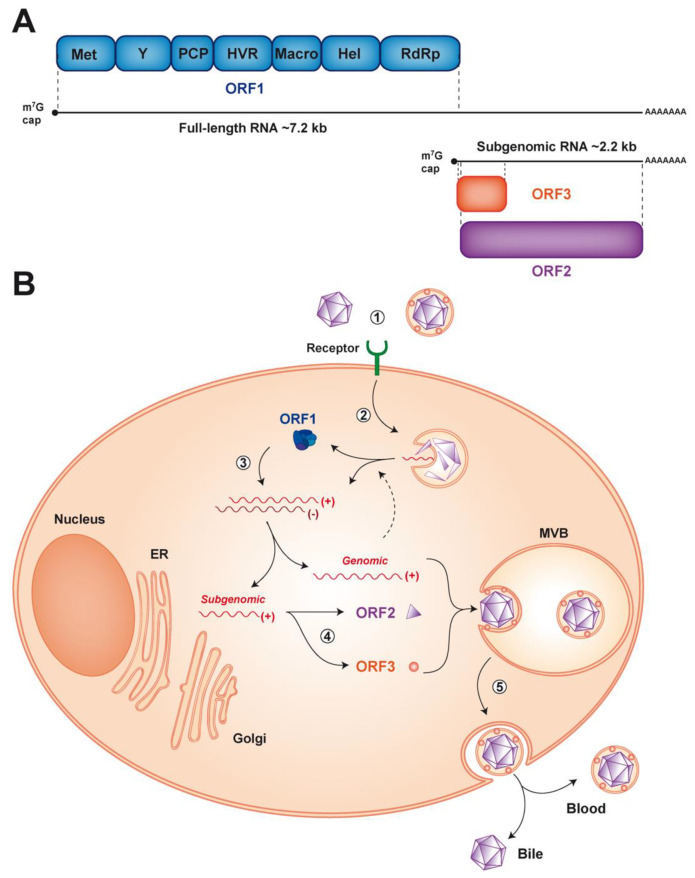
Genome organization and life cycle of hepatitis E virus (HEV). (**A**) The 7.2 kb positive-strand RNA genome has a 5′ 7-methylguanylate cap (m^7^G cap) and a 3′ polyadenylated tail (poly-A). It harbors 3 open reading frames (ORFs). ORF1 encodes a replicase of about 190 kDa comprising different functional domains, including a methyltransferase (Met), an RNA helicase (Hel) and an RNA-dependent RNA polymerase (RdRp), as well as less well-characterized domains, such as the Y domain, a putative papain-like cysteine protease (PCP), a hypervariable region (HVR) and the Macro domain. ORF2 and ORF3 encode the viral capsid and a small protein involved in virus secretion respectively, which are translated from a 2.2 kb subgenomic RNA generated during viral replication. (**B**) The HEV life cycle can be dissected into the following steps: (1) viral entry by as yet unidentified receptor(s), (2) endocytosis and release of the viral positive-strand RNA genome (+) into the cytosol, (3) translation of the ORF1 protein to allow replication of the full-length and generation of the subgenomic RNA through a negative-strand RNA intermediate (-), (4) translation of the subgenomic RNA to produce the ORF2 and ORF3 proteins and (5) genome packaging, virion assembly and release of the virus into the bloodstream and the bile from the basolateral and apical sides, respectively. ER, endoplasmic reticulum; MVB, multivesicular body.

HEV is a small, non-enveloped, icosahedral virus with a diameter of 27–34 nm [2]. It contains a 7.2 kb single-stranded, positive-sense RNA genome which possesses a m7G cap at its 5′ and a poly-A tail at its 3′ end (Figure 1A). The HEV genome harbors 3 open reading frames (ORF). ORF1 encodes the viral replicase, ORF2 the capsid and ORF3 a small protein involved in virion secretion via its potential ion channel activity [12]. The first contact between HEV and host cells occurs through interaction with as yet poorly characterized entry factor(s). After endocytosis, the viral genome is released into the cytoplasm and the host translational machinery produces the ORF1 replicase, which drives viral RNA replication (Figure 1B). During this step, two RNA species are produced from a negative-strand RNA intermediate: a full-length genomic RNA and a subgenomic RNA of 2.2 kb [13,14]. Translation of the subgenomic RNA yields the ORF2 and ORF3 proteins. Later steps of the HEV life cycle include viral assembly and release of newly produced virions. Very similar to hepatitis A virus (HAV), another hepatotropic positive-strand RNA virus, HEV is found as a ‘quasi-enveloped’ virion (eHEV) wrapped in exosomal membranes in blood and as a naked particle in bile and feces (reviewed in [15]) (Figure 1B).

As obligate intracellular pathogens, viruses have developed strategies to hijack and manipulate host cell pathways in order to ensure productive infection. Moreover, RNA viruses, especially those with relatively limited genome size and coding capacity, such as HEV, are particularly dependent on the host cell machinery. Because the tools to study HEV have been limited until recently, only little is known about the host factors involved in the various steps of the viral life cycle. Studies performed in heterologous settings, such as the yeast two hybrid system, identified cellular factors interacting with HEV proteins [16,17]. However, most of these candidates remain to be validated and further studied using infectious cell culture systems, in vivo models and liver biopsies from patients with hepatitis E. In this review, we shall focus on host factors whose involvement in the viral life cycle has been validated in HEV infection settings.

## 2. HEV Entry

**Figure 2:**
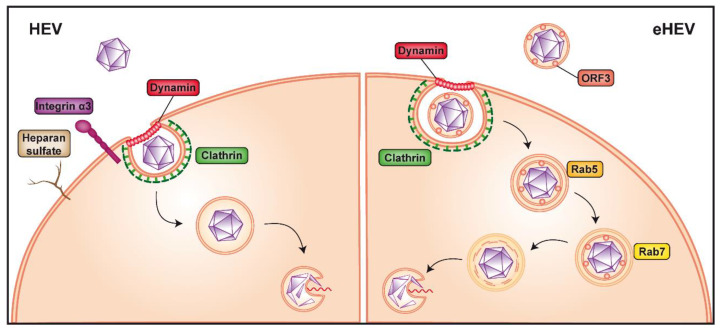
Non-enveloped versus quasi-enveloped hepatitis E virus entry. Non-enveloped hepatitis E virus (HEV) is believed to first bind to heparan sulfate proteoglycans and integrin α3 at the cell surface (left panel). The virus is then internalized via clathrin- and dynamin 2-dependent endocytosis, followed by release of the viral genome into the cytoplasm by an as yet unknown mechanism, possibly involving a conformational change of the capsid. The cofactor(s) and receptor(s) allowing entry of quasi-enveloped HEV (eHEV) are unknown (right panel). Internalization requires clathrin- and dynamin 2-dependent endocytosis as well as trafficking through Rab5- (early) as well as Rab7-positive (late) endosomes and eventually lysosomes to allow release of the viral genome into the cytoplasm, likely by a process similar to that of naked HEV.

As HEV is present in a non-enveloped ("naked") and a quasi-enveloped form (eHEV), the entry pathway of the virus may differ for these two forms. Our knowledge of HEV entry remains scarce but studies using virus-like particles (VLP) as a model system have highlighted possible host factors involved in the initial attachment to the cell and virus internalization, including the 78-kDa glucose-regulated protein (GRP78), ATP synthase subunit β (ATPB5) and asialoglycoprotein receptor (ASGPR) [18,19,20]. Notably, non-enveloped HEV was shown to interact with heparan sulfate proteoglycans (HSPG), likely syndecans [21,22], which are expressed on the surface of many cell types. Hence, treatment of susceptible hepatoma cell lines with heparinase I considerably reduced VLP binding as well as HEV infection [21] (Figure 2). Of note, HSPG are known to mediate cell attachment of several enveloped and nonenveloped viruses, including, among others, herpes simplex virus, hepatitis C virus (HCV), norovirus and human immunodeficiency virus [23,24,25,26,27].

A recent microarray analysis comparing gene expression in permissive versus non-permissive cells identified integrin α3 as an entry factor for HEV [28]. Integrins belong to a family of transmembrane proteins localized at the cell surface which can bind to extracellular matrix (ECM) as well as cell surface and intracellular ligands. These interactions trigger a myriad of intracellular signals modulating cell behavior through effects on actin microfilaments, thereby connecting the extracellular space and the cytoskeleton (reviewed in [29]). Integrins were shown to function as receptors for Kaposi’s sarcoma-associated herpesvirus [30] and, interestingly, also for HAV [31]. In the case of HEV, overexpression of integrin α3 in non-permissive cells allowed non-enveloped HEV infection only and, conversely, knockout of integrin α3 gene in permissive cells prevented entry of non-enveloped HEV but not of eHEV [28]. Together with the physical interaction between integrin α3 and HEV, the data strongly suggests that this ECM receptor is an entry factor for the non-enveloped viral particle (Figure 2). Integrins are expressed in a broad array of tissues, including in the intestine [26,32], where HEV is known to replicate and which may represent the initial site of infection [33,34]. However, in some organs, such as the liver, integrin α3 expression is rather low [35,36,37], raising the possibility that integrin α3 may act as a cofactor for HEV entry rather than as the key receptor. Further studies are needed to clarify the entry pathway used by non-enveloped HEV.

Internalization of non-enveloped HEV is believed to occur through clathrin- and dynamin 2-dependent endocytosis [22,38,39] (Figure 2). In this context, membrane cholesterol was also shown to be important for HEV entry, as treatment of cells with cholesterol sequestering agents significantly reduced VLP uptake [39]. Following endocytosis, the HEV genome needs to be uncoated to be released into the cytoplasm. It is likely that the capsid protein itself plays a crucial role in this poorly understood process, possibly by undergoing conformational changes induced by interaction with a host protein, as it is seen in other non-enveloped viruses, such as human papilloma virus, murine polyomavirus and poliovirus [40,41,42].

Since the viral capsid is not exposed at the surface of eHEV (see Section 4), the quasi-enveloped particle may use different, as yet unknown entry factor(s). However, as for the naked virus, eHEV is internalized through clathrin- and dynamin 2-dependent endocytosis [38,39]. Unlike non-enveloped HEV, eHEV entry is dependent on the small GTPases, Rab5 and Rab7, both of which are involved in endosomal trafficking. In fact, depletion of one or the other has been shown to considerably reduce virus infection [22] (Figure 2). In addition, eHEV entry depends on endosomal acidification [22], indicating that transition of the endosome to a more acidic cell compartment is necessary (Figure 2). Lysosomal lipid degradation appears to be a required step in eHEV entry. Indeed, depletion of the late endosomal and lysosomal Niemann-Pick disease type C1 protein, involved in cholesterol extraction, significantly reduced eHEV infection [22]. Moreover, treatment with an inhibitor of lysosomal acid lipase, responsible for lipid degradation, resulted in a dose-dependent reduction of eHEV cell entry [22].

These observations suggest that the quasi-envelope of eHEV is removed in endolysosomes. The viral capsid may subsequently interact with an as yet unidentified host factor and undergo the conformational changes required for genome release into the cytoplasm. A similar mechanism was recently proposed for HAV [31]. Since both quasi-enveloped and non-enveloped HEV are internalized in vesicles belonging to the endosomal pathway, it is plausible that they use a common host factor to allow uncoating and release of the genome into the cytoplasm (detailed in [43]).

The ORF3 protein is present within eHEV and interacts with the capsid [44]. However, its role in eHEV entry, uncoating and genome release remains to be explored.

Taken together, some HEV entry factors and pathways have been identified, but key elements are still missing. The capacity of HEV to infect a broad array of cell types [45,46,47,48,49,50,51], possibly related to the diverse extrahepatic manifestations of hepatitis E [52,53,54,55], strongly suggests that the entry receptor(s) is (are) not specific to hepatocytes but ubiquitously expressed.

## 3. Viral RNA Replication

Upon release of the viral positive-strand RNA genome into the cytoplasm, the host translational machinery, including ribosomal subunits and elongation factors, seeds on the 5′ untranslated region to start translation of the ORF1 protein. Among the different components, the eukaryotic translation initiation factor 4F (eIF4F) complex has been identified as being important for HEV replication. This complex is known to be involved in the cap-dependent translation and replication of several viruses (reviewed in [56]). In this context, an RNA interference-based loss-of-function study showed that components of the eIF4F complex are required for efficient HEV replication, while known negative regulatory factors of this pathway limit viral RNA synthesis [57]. Along these lines, silvestrol, a natural compound inhibiting part of the eIF4F complex, efficiently inhibits HEV replication in vitro, and in a mouse model in vivo, ORF2 protein production and virus spread [58,59]. Interestingly, silvestrol was reported to also inhibit corona-, picorna-, alpha-, flavi- and filo-viruses and, therefore, displays broad antiviral activity [60,61,62,63].

ORF1 encodes the functional domains required for viral RNA synthesis, including an RNA-dependent RNA polymerase (RdRp) located at the *C*-terminal end of the polyprotein. In addition to the RdRp, the replicase encoded by ORF1 comprises an RNA helicase, a methyltransferase as well as less well-characterized domains, including the Macro and Y domains, a hypervariable region and a putative papain-like cysteine protease (PCP) (Figure 1A). Of note, the PCP has been associated with deubiquitination and the Macro domain with deribosylation activities likely involved in the posttranslational modifications of host proteins [64,65]. Positive-strand RNA viruses commonly process their polyproteins into individual functional proteins by viral, and in some instances, cellular proteases. In the case of HEV, however, it is unsettled whether, and if so, by which mechanism, the ORF1 protein is processed. While some studies reported processing of the ORF1 polyprotein, including by the PCP or the cellular proteases thrombin and factor Xa [66], others have found that the major form of ORF1 protein is unprocessed (reviewed in [53,67]). Further studies in complete cell culture systems and using more sensitive techniques for the detection of ORF1 protein are required [68].

As for all positive-strand RNA viruses, replication includes synthesis of a complementary negative-strand RNA which serves as a template for the production of positive-strand genomic and, in the case of HEV, an additional subgenomic RNA. The key enzyme responsible for these steps is the RdRp. The mechanisms regulating the production of these RNA species from the negative-strand RNA are still unknown but imply cis-acting elements within the genome [69]. Regulation of transcription may also involve host factor(s), such as heterogeneous nuclear ribonucleoproteins (hnRNP) [70,71]. hnRNPs play a role in nuclear RNA metabolism and have been reported to re-localize to the cytoplasm of HEV-infected cells [71], similarly to what had been observed for infection with other RNA viruses [72,73,74,75,76], suggesting a potential role of hnRNPs in genomic and subgenomic HEV RNA transcription.

Replication of positive-strand RNA viruses takes place in membrane-associated replication complexes composed of viral proteins, the replicating viral RNA, rearranged cellular membranes and other host factors [77]. The subcellular site of HEV RNA replication has not been identified to date but recent advances such as the development of tagged functional HEV genomes may facilitate progress in this area [68]. Insertion of a hemagglutinin epitope tag in the ORF1 polyprotein allowed us to visualize the HEV replicase together with viral RNA in cytoplasmic dot-like structures, likely indicating the site of active replication. ORF1 protein was found to colocalize best with exosomal markers as well as with the ORF2 and ORF3 proteins, suggesting that HEV RNA replication takes place in close proximity to virion assembly sites [68]. Virus-induced membrane rearrangements may serve several purposes, including the physical support and organization of the replication complex, the compartmentalization and local concentration of viral and host factors required for RNA replication, tethering of the viral RNA during unwinding and coordination of its translation, replication and packaging, provision of lipid constituents important for replication, and physical protection from host antiviral defenses (reviewed in [77,78]). Of note, HCV as well as picorna- and coronaviruses require the guanine nucleotide-exchange factor Golgi brefeldin A resistance factor 1 (GBF1) for the induction of membrane alterations making up their viral replication complexes [79,80,81,82,83]. Interestingly, similar observations have been made for HEV [84]. However, no colocalization of GBF1 with HEV ORF1 and ORF2 proteins or relocalization of GBF1 upon HEV infection have been observed. One may thus hypothesize that GBF1 is not recruited to HEV replication sites and is not involved in replication complex formation but rather plays an indirect role in HEV replication [84], as also proposed for HCV [79,80] and mouse hepatitis virus [85].

## 4. Virion Assembly and Infectious Particle Release

Virion assembly consists of the packaging of genomic viral RNA in the capsid. While the subcellular site of HEV assembly has not been identified yet, it is likely tightly connected to replication complexes (see above). The mechanisms driving HEV assembly are poorly understood but early observations showed that RNA and the ORF2 protein can spontaneously assemble into virus-like particles in insect cells [86] (reviewed in [87]). These findings argue in favor of a self-assembly process involving a limited number of host factors.

The ORF2 protein exists in several forms, of which a non-glycosylated form is involved in virion formation [88,89,90,91]. It is currently debated whether this form, harboring a truncated and non-functional signal peptide, results from translation at an alternative start site [90] or from proteolytic cleavage, as suggested by mass spectrometry analyses [89]. The non-glycosylated ORF2 protein may represent the major intracellular form detected in the cytosol as well as the cell nucleus [89,91,92]. While the role of nuclear ORF2 protein is unknown, its nucleocytoplasmic shuttling likely involves the host nuclear import/export machineries. In contrast to the non-glycosylated form packaging the viral genome, the glycosylated form of ORF2 protein is rapidly released extracellularly through the secretory pathway [88,89,90,91,93,94]. This implies recognition of its signal peptide by the translocon, translocation into the ER lumen, cleavage by signal peptidase, followed by sialylation as well as *N*- and *O*-glycosylation by yet to be defined glycosyltransferases in the ER and Golgi [89], and secretion. Glycosylated ORF2 protein is present in at least two forms, of which the smaller may result from cleavage by furin-like proteases at an RRR motif [91]. Overall, these studies suggest that ORF2 protein likely has more than one function in genome packaging and point toward the implication of different host factors.

**Figure 3:**
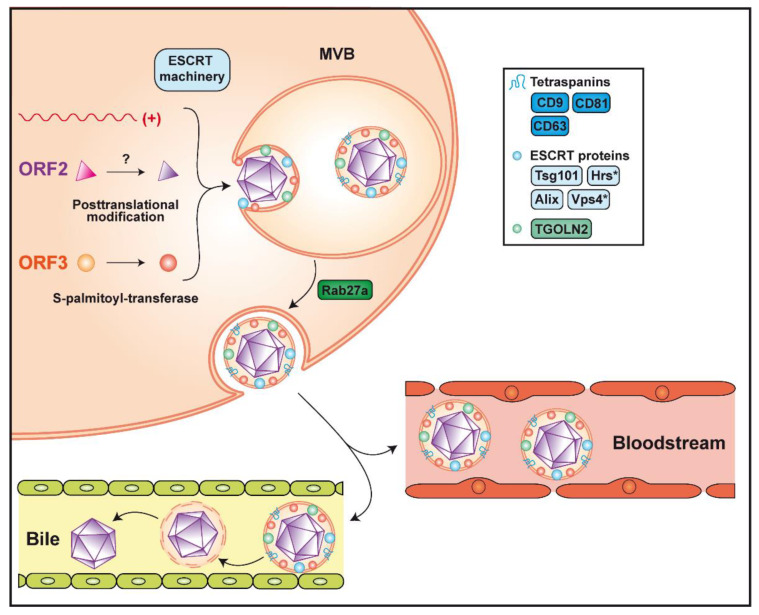
Assembly and release of infectious hepatitis E virus. Packaging of the viral genome into the capsid is believed to occur by spontaneous self-assembly of the non-glycosylated ORF2 protein. The non-glycosylated ORF2 protein may undergo post-translational modification by yet unknown enzyme(s). Formation of the quasi-enveloped particle involves phosphorylation and palmitoylation of the ORF3 protein and the ESCRT machinery, of which the components Tsg101, Hrs, Vps4 and Alix were shown to be required (see text for abbreviations). The virion is wrapped in an exosomal membrane harboring the tetraspanins CD9, CD63 and CD81, as well as the trans-Golgi network protein 2 (TGOLN2), Alix and Tsg101. Release of quasi-enveloped HEV (eHEV) involves Rab27a-dependent trafficking of multivesicular bodies (MVB) and fusion with the plasma membrane. Secreted particles remain associated with the lipid membrane in the bloodstream while they are delipidated in the bile. Asterisks indicate host factors that were not found on the quasi-envelope of eHEV.

HEV assembly involves the non-glycosylated ORF2 protein and the viral RNA but does not require the ORF3 protein, as genomes harboring a mutated ORF3 start codon yield infectious particles; however, these are not secreted from the cell [95,96]. Infectious virus is believed to be released as quasi-enveloped particles from both the basolateral and apical sides of hepatocytes, facing the liver sinusoids and the bile canaliculi, respectively [33,97,98]. The pseudo-envelope of virions secreted into the bile is likely delipidated by bile acids and/or pancreatic enzymes (Figure 3). Interestingly, HEV is preferentially secreted from the apical side, explaining the high viral load detected in feces. Based on these observations, different host factors may be involved in virus secretion from the apical and basolateral sides of hepatocytes. Moreover, HEV ORF3 protein is essential for the secretion of infectious particles, possibly by connecting the capsid with the host factors required for egress. Phosphorylation of ORF3 protein at a serine residue (Ser 70 and Ser 71 in gt 3 and gt 1, respectively) may trigger the interaction with assembled non-glycosylated ORF2 protein [44]. Based on sequence information, phosphorylation may involve the p34cdc2 kinase and mitogen-activated protein kinase [99]. Furthermore, ORF3 protein recruits Tsg101, a member of the endosomal sorting complex required for transport-I (ESCRT-I), via a highly conserved PSAP motif [100,101,102]. The presence of this motif in the *C*-terminal region of the ORF3 protein is required for virus release [101]. It is known that proline-rich motifs P(S/T)AP and PPXY (X being any amino acid), which are also called "late domains", are essential for budding of enveloped viruses such as human immunodeficiency virus (HIV)-1 or Ebola [103,104]. The ESCRT machinery is composed of 4 complexes, ESCRT-0 to ESCRT-III, which are involved in membrane remodeling, leading to budding reactions or membrane involution. These complexes can further recruit accessory proteins, such as vacuolar protein sorting 4 (Vps4) and apoptosis-linked gene-2 interacting protein X (Alix), both interacting with the ESCRT-III complex, to close newly formed vesicles (reviewed in [105]). Further confirming the requirement for the ESCRT machinery, hepatocyte growth factor-regulated tyrosine kinase substrate (Hrs), a member of ESCRT-0, and Vps4 were shown to be required for HEV particle release [102,106]. In addition, the envelope wrapping the virion has been shown to be derived from exosomes, small vesicles generated from multivesicular bodies (MVB) by the ESCRT pathway [102,106]. These MVB, which fuse with the plasma membrane to release their content into the extracellular milieu, require the Rab27a protein, which has been shown to colocalize with ORF3 protein [102,106] (Figure 3).

Current evidence indicates that none of the viral proteins are present on the surface of eHEV but identified ORF3 protein beneath the quasi-envelope [107,108,109]. Palmitoylation at *N*-terminal cysteine residues mediates membrane association as well as stability of the ORF3 protein and is required for virus secretion [110]. A membrane topology where ORF3 protein is located entirely on the cytosolic side, corresponding to the exosome lumen, has therefore been proposed [110] and is further supported by the interaction of its PSAP motif with Tsg101. Of note, palmitoylation is a reversible protein modification taking place in the cytosol, which increases the hydrophobicity of a protein and contributes to its membrane association (reviewed in [111]). This implies the requirement for one or more as yet unidentified *S*-palmitoyl-transferase(s) of the host cell [110].

Given the origin of the quasi-envelope, host proteins, and more specifically, exosomal proteins, can be present on eHEV. Indeed, particles released from infected cells and positive for ORF2 and ORF3 proteins were shown to harbor classical exosomal markers, including the tetraspanins CD81, CD63 and CD9, as well as the ESCRT components Alix and Tsg101 [106,108,112]. Interestingly, trans-Golgi network protein 2 (TGOLN2) has also been detected on eHEV [107] (Figure 3). This observation and the fact that TGOLN2 is located in the cytoplasm confirms that quasi-enveloped particles are produced within the cell and not at the plasma membrane.

The quasi-enveloped nature of virions circulating in blood may provide several advantages to HEV, including protection from neutralizing antibodies. However, the presence of eHEV in blood may also favor virus dissemination to organs other than the liver, as exosomes containing viral genetic material were shown to lead to productive infection by other viruses and to modulate cellular responses (reviewed in [113]). Hence, it is plausible that the production of quasi-enveloped virions has additional functions beyond the spread of HEV within the liver.

## 5. Conclusions and Perspectives

Although HEV research is a rapidly growing field, our current knowledge of the virus life cycle is still limited by important gaps. We lack key information on virus entry, including the cellular receptor(s), and on the uncoating of the viral RNA. Moreover, future efforts should concentrate on the molecular mechanisms and subcellular compartments involved in RNA replication and assembly. While virus release remains one of the best studied steps of the viral life cycle, many aspects need to be clarified, in particular the contribution of host factors and the composition and role of quasi-enveloped particles. Considering the ability of HEV to replicate in different tissues as well as to infect a wide range of animals, one may hypothesize that HEV host dependency is not very selective, facilitating the crossing of species barriers.

Studies on the HEV life cycle currently rely on the use of in vitro model systems, which have certain limitations, especially with respect to host factors present in differentiated hepatocytes. As an example, a stimulatory effect of cyclophilin inhibitors on HEV replication was reported initially in hepatoma cells [114]. However, in stem cell-derived hepatocyte-like cells, this observation was confirmed only with a cell culture-adapted infectious clone, but not with natural HEV isolates [115]. Future improvements of in vitro models should include the use of natural HEV isolates together with primary and stem cell-derived hepatocyte-like cells, polarized cell culture models, as well as ex vivo and in vivo infection model systems to confirm in vitro findings. Ultimately, key findings will have to be validated in liver specimens from patients with hepatitis E.

Obtaining a broader and unbiased view of the host factors involved in the HEV life cycle will likely depend on novel technologies, such as clustered regularly interspaced short palindromic repeats (CRISPR)/Cas9-based genome-wide screening and proteomic proximity labeling approaches. CRISPR/Cas9-based screens have advanced the understanding of, among others, virus entry [116,117] and replication [118,119], and have also facilitated the identification of new antiviral targets [120]. Proximity labeling has been successfully used, for example, to characterize the microenvironment of coronavirus replication complexes [121]. In the future, the combination of improved HEV model systems and of novel technologies should improve our knowledge of the host factors required for productive HEV infection.
